# Decoding the Role of Sphingosine-1-Phosphate in Asthma and Other Respiratory System Diseases Using Next Generation Knowledge Discovery Platforms Coupled With Luminex Multiple Analyte Profiling Technology

**DOI:** 10.3389/fcell.2020.00444

**Published:** 2020-06-19

**Authors:** Sami Bahlas, Laila A. Damiati, Ayman S. Al-Hazmi, Peter Natesan Pushparaj

**Affiliations:** ^1^Department of Internal Medicine, Faculty of Medicine, King Abdulaziz University, Jeddah, Saudi Arabia; ^2^Department of Biology, Faculty of Biological Sciences, University of Jeddah, Jeddah, Saudi Arabia; ^3^Department of Clinical Laboratory Sciences, College of Applied Medical Sciences, Taif University, Makkah, Saudi Arabia; ^4^Department of Medical Laboratory Technology, Faculty of Applied Medical Sciences, King Abdulaziz University, Jeddah, Saudi Arabia; ^5^Center of Excellence in Genomic Medicine Research, Faculty of Applied Medical Sciences, King Abdulaziz University, Jeddah, Saudi Arabia

**Keywords:** asthma, respiratory diseases, sphingosine-1-phosphate, SwissTargetPrediction, WebGestalt, open targets platform, ingenuity pathway analysis, Luminex xMAP technology

## Abstract

Sphingosine-1-phosphate (S1P) is a pleiotropic sphingolipid derived by the phosphorylation of sphingosine either by sphingosine kinase 1 (SPHK1) or SPHK2. Importantly, S1P acts through five different types of G-protein coupled S1P receptors (S1PRs) in immune cells to elicit inflammation and other immunological processes by enhancing the production of various cytokines, chemokines, and growth factors. The airway inflammation in asthma and other respiratory diseases is augmented by the activation of immune cells and the induction of T-helper cell type 2 (Th2)-associated cytokines and chemokines. Therefore, studying the S1P mediated signaling in airway inflammation is crucial to formulate effective treatment and management strategies for asthma and other respiratory diseases. The central aim of this study is to characterize the molecular targets induced through the S1P/S1PR axis and dissect the therapeutic importance of this key axis in asthma, airway inflammation, and other related respiratory diseases. To achieve this, we have adopted both high throughput next-generation knowledge discovery platforms such as SwissTargetPrediction, WebGestalt, Open Targets Platform, and Ingenuity Pathway Analysis (Qiagen, United States) to delineate the molecular targets of S1P and further validated the upstream regulators of S1P signaling using cutting edge multiple analyte profiling (xMAP) technology (Luminex Corporation, United States) to define the importance of S1P signaling in asthma and other respiratory diseases in humans.

## Introduction

Sphingosine-1-phosphate (S1P), a sphingolipid, is one of the essential modulators of various cellular processes such as differentiation, survival, growth, etc. (Aarthi et al., 2011). Sphingolipids are recently termed as “morphogenetic lipids” for their crucial role in embryonic stem cell differentiation, survival, and postnatal development (Wang et al., 2018). It was recently demonstrated that S1P plays an important role in allergic anaphylaxis and asthma (Schauberger et al., 2016; Kim, 2019). Asthma is a heterogeneous and complex disease typically characterized by bronchial hyperactivity, remodeling of lung tissues, and chronic bronchial inflammation (Mims, 2015). Importantly, asthma is predisposed by both genetic and environmental factors and the incidence of Asthma has been ever increasing in both children and adults around the globe (Beran et al., 2015; McGeachie et al., 2015). Approximately 1000 people die each day due to Asthma and there were 339.4 million people affected by asthma globally in 2016. This represents a 3.6% increase in age-standardized prevalence since 2006 (Global Asthma Network, 2018; Global Initiative for Asthma, 2018). Asthma affects individuals of all ages and all ethnicity and the economic burden of respiratory diseases to governments, healthcare systems, families, and patients are on the rise globally (Singh, 2005; Ellwood et al., 2017). Hence, creating new applied scientific knowledge in the area of asthma and related respiratory diseases has become a key priority around the globe (Ellwood et al., 2017; Koshak et al., 2017).

S1P is the ligand for G-protein coupled receptors (GPCRs) that belong to the Endothelial Differentiation Gene (EDG) family of proteins termed as S1P receptors (S1PRs) (van Koppen et al., 1996; Hla, 2001; Spiegel and Milstien, 2003; Aarthi et al., 2011). S1P can specifically stimulate distinct genetic events depending on the relative expression of S1PRs as well as downstream G-proteins (An et al., 1998; Aarthi et al., 2011; Manikandan et al., 2012). The intracellular S1P is transported through ATP binding cassette transporter, ABCC1 to the extracellular milieu (Mitra et al., 2006; Nieuwenhuis et al., 2009) and causes the chemotaxis of inflammatory cells that is a pre-requisite for *T*-lymphocyte exit from primary and secondary lymph organs (Aarthi et al., 2011). It was shown that the S1P/S1PR axis is important for the initiation of various pathophysiological processes that possibly leading to critical diseases such as cancer, inflammatory diseases, and disorders in humans and other higher eukaryotes (Aarthi et al., 2011). Recently, FDA has approved the first oral drug, named Gilenya (FTY720 or Fingolimod), a sphingosine analog for the treatment of relapse in Multiple Sclerosis (MS) (Aarthi et al., 2011). The sphingolipid metabolism was significantly affected in asthma and the S1P levels were increased and correlated with the severity of the disease (Mohammed and Harikumar, 2017; Saluja et al., 2017). The clinical phenotypes of asthma with distinct disease mechanisms are critically influenced by levels of sphingolipid metabolites such as S1P (McGeachie et al., 2015). It was shown that the changes in sphingolipid metabolism and airway hyperresponsiveness were positively correlated in patients suffering from dust mite allergy and the intravascular levels of both sphinganine-1-phosphate (SA1P) and S1P, were significantly associated with the severity of bronchial hyperreactivity (Kowal et al., 2019). The phenotypes of asthma may be distinct based on the levels of sphingolipid metabolites and the change in sphingolipids could represent a pathophysiological variation in bronchial hypersensitivity to allergens in asthmatics (McGeachie et al., 2015).

To address this important problem, we have designed this study to delineate the functions of S1P in the development of asthma and other respiratory diseases. Here, we have utilized both next-generation knowledge discovery platforms such as SwissTargetPrediction, WebGestalt, Open Targets Platform, and Ingenuity Pathway Analysis (Qiagen, United States) to delineate the molecular targets of S1P and validated the upstream regulators of S1P signaling using cutting edge multiple analyte profiling (xMAP) technology (Luminex Corporation, United States) (Bahlas et al., 2019; Pushparaj, 2019; Harakeh et al., 2020; Jafri et al., 2020).

## Materials and Methods

### SwissTargetPrediction Analysis

The *in silico* prediction of the molecular targets of S1P was performed using Swiss Target Prediction, a virtual screening web tool (Gfeller et al., 2013). Both canonical and isomeric SMILES of S1P were used as an input sequence as described before (Daina et al., 2019). In the SwissTargetPrediction web tool, the similarity principle is used to predict the targets by reverse screening strategy. In the SwissTargetPrediction web tool, the predictions are performed from analogous molecules in 2D and 3D, from 376342 experimentally active compounds that are significantly interacting with 3068 known macromolecular targets (Daina and Zoete, 2019).

### WebGestalt Analysis of S1P Targets

The Over Representation Analysis (ORA) of the molecular targets of S1P derived using Swiss Target Prediction was used in the WebGestalt tool (wGSEA) (Zhang et al., 2005; Wang et al., 2013, 2017; Liao et al., 2019). Using wGSEA gene lists obtained from large scale -omics studies were categorized based on biological, molecular, and cellular functions. wGSEA is a freely available open-source platform^1^ that enables a more broad, effective, flexible, and interactive functional enrichment study (Liao et al., 2019). The latest version of the wGSEA recognizes 155175 functional categories, 342 gene identifiers, and 12 organisms including many user-defined functional databases (Liao et al., 2019).

To functionally characterize the S1P-induced molecular profile, we analyzed the molecular targets of S1P obtained by Swiss Target Prediction using ORA (Liao et al., 209). Here, the enrichment method was chosen as ORA, the selected organism was Homo sapiens, and gene ontology (Molecular Function, Biological Function, and Cellular Function) was selected for each type of analysis. The reference list for each analysis included all mapped Entrez gene IDs from the selected platform genome. The parameters for the enrichment analysis included the minimum number of IDs in the category (5), the maximum number of IDs in the category (2000), the Benjamini Hochberg (BH) method (*P* < 0.05) for computing the False Discovery Rate (FDR) (*P* < 0.05), and the significance Level (Top 10).

### Open Targets Platforms Analysis

The Open Targets Platform was used to find the S1P molecular targets associated with respiratory diseases (Koscielny et al., 2017; Carvalho-Silva et al., 2019; Zhang et al., 2019). The evidence from scientific literature, animal models, genomics, transcriptomics, genetics, and drugs are used in the Open Targets Platform to score and rank target-disease associations and aid target prioritization (Carvalho-Silva et al., 2019; Zhang et al., 2019). The query list with 93 molecular targets of S1P was used to find the respiratory diseases significantly (*P* < 0.05) regulated by the S1P signaling and its associated molecular networks.

### Ingenuity Pathway Analysis

Ingenuity pathway analysis (IPA) software has a cutting edge up to date next generation knowledge base consists of clarified scientific information from publications, databases, and other relevant resources (Jafri et al., 2020). Here, we applied the IPA software (Qiagen, United States) to functionally annotate the gene clusters and identified biologically significant pathways regulated by S1P. The molecular target of S1P was subjected to Core Analysis in the IPA to delineate biologically relevant canonical pathways as well as novel molecular signatures, using the right-tailed Fisher Exact Test and Benjamini Hochberg Correction (BHC) for multiple testing (*P* < 0.05), affecting the respiratory diseases through S1P/SPHK pathway to deduce unique disease-causing gene clusters.

### Luminex xMAP Assay for Biomarkers of Asthma

The blood samples were collected from healthy volunteers and asthma patients and both groups were age, and sex-matched. Healthy non-atopic individuals (*n* = 12) were used as control and the asthma patients (*n* = 12) were with moderate to severe disease state, non-smokers, and without any other co-morbid conditions such as chronic obstructive pulmonary disease (COPD), other types of pulmonary diseases and disorders, diabetes, cancer, autoimmune diseases, etc. The institutional ethical approval was obtained before collecting the samples and the study was approved by the Scientific Research Committee, Deanship of Scientific Research (DSR), King Abdulaziz University (KAU), Jeddah. The peripheral blood isolated was kept at room temperature for clotting and then centrifuged at 3000 rpm for 10 min for the separation of serum and the aliquots were stored at −80°C till the xMAP assays were done (Bahlas et al., 2019). The evaluation of cytokines and growth factors filtered using the next-generation knowledge discovery platforms was done in both healthy controls and asthma patients using a multi-cytokine/chemokine (30-plex) magnetic-bead based fluorescence assay and the results were obtained based on the standard curves generated using the 30plex standards (16 plex and 14 plex vials) supplied with the xMAP kit (Catalog No: LHC6003M) (Novex, Invitrogen, United States) using MAGPIX multiplex platform as described before (Bahlas et al., 2019; Pushparaj, 2019; Harakeh et al., 2020; Jafri et al., 2020; Pushparaj, 2020). Serum samples were also used to evaluate the concentration of S1P in both healthy controls and asthma patients using a competitive ELISA method as described before (Milara et al., 2012). The absorbance was measured at 450 nm using SpectraMax i3 Multi-Mode Reader with MiniMax Imaging Cytometer (Molecular Devices, United States).

### Statistical Analyses

The raw xMAP data was analyzed by the xPONENT analysis software (Version 4.2) (Luminex Corporation, Austin, TX, United States) to determine the absolute concentration of cytokines and growth factors in both healthy control and asthma groups (Bahlas et al., 2019; Harakeh et al., 2020; Jafri et al., 2020). The statistical significance was further computed by using GraphPad Prism Version 8.3 (GraphPad Software, San Diego, CA, United States). *P* ≤ 0.05 was considered to be statistically significant based on the Student’s *t*-test (Two Tail). The values were represented as mean ± SD. Furthermore, an *F*-test to compare the variances and simple correlation analysis of S1P levels against cytokines and chemokines in the serum of asthma patients was also performed using GraphPad Prism (Version 8.3) software.

## Results

### *In silico* Prediction of the Molecular Targets of S1P Using SwissTargetPrediction

**FIGURE 1:**
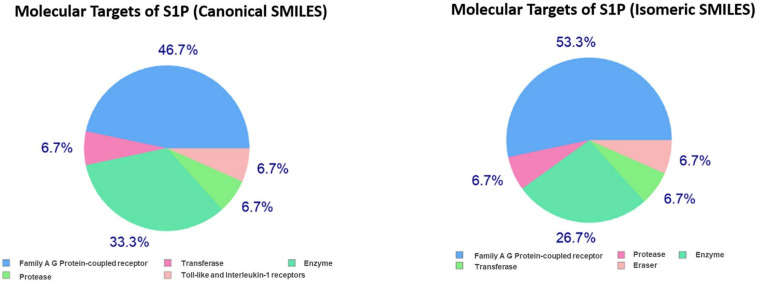
Swiss Target Prediction for sphingosine-1-phosphate (S1P). Top 15 percent of the molecular targets (Homo sapiens) of S1P obtained using both canonical and isomeric Simplified Molecular Input Line Entry System (SMILES) codes in Swiss Target Prediction server.

In the present study, the SwissTargetPrediction was performed for S1P (C18H38NO5P) using both Canonical [CCCCCCCCCCCCCC = CC(C(COP(= O)(O)O)N)O] and Isomeric (CCCCCCCCCCCCC/C = C/[C@H]([C@H](COP(= O) (O)O)N)O) Simplified Molecular Input Line Entry System (SMILES) codes (Supplementary Figure S1) computed by OEChem (Version 2.1.5), have shown that it interacts with 64 and 93 molecules respectively (Tables 1A,B) with the highest percentage of binding (46.7 and 53.3%, respectively) with Family A GPCRs (Figure 1).

**TABLE 1A:** SwissTargePrediction of the molecular targets of S1P (Canonical SMILES) in Homo sapiens.

**Target**	**Common name**	**Uniprot ID**	**ChEMBL ID**	**Target class**	**Probability**	**Known actives (3D/2D)**
Sphingosine 1-phosphate receptor Edg-8	S1PR5	Q9H228	CHEMBL2274	Family A G protein-coupled receptor	0.843606914	42/1
Sphingosine 1-phosphate receptor Edg-6	S1PR4	O95977	CHEMBL3230	Family A G protein-coupled receptor	0.843606914	40/1
Sphingosine 1-phosphate receptor Edg-3	S1PR3	Q99500	CHEMBL3892	Family A G protein-coupled receptor	0.843606914	95/2
Sphingosine 1-phosphate receptor Edg-1	S1PR1	P21453	CHEMBL4333	Family A G protein-coupled receptor	0.843606914	123/2
Sphingosine 1-phosphate receptor Edg-5	S1PR2	O95136	CHEMBL2955	Family A G protein-coupled receptor	0.783474129	10/1
Lysophosphatidic acid receptor Edg-7	LPAR3	Q9UBY5	CHEMBL3250	Family A G protein-coupled receptor	0.097239989	12/10
Farnesyl diphosphate synthase	FDPS	P14324	CHEMBL1782	Transferase	0	11/0
Sphingosine kinase 2	SPHK2	Q9NRA0	CHEMBL3023	Enzyme	0	0/2
Sphingosine kinase 1	SPHK1	Q9NYA1	CHEMBL4394	Enzyme	0	0/3
Lysophosphatidic acid receptor Edg-2	LPAR1	Q92633	CHEMBL3819	Family A G protein-coupled receptor	0	10/5
Squalene synthetase (by homology)	FDFT1	P37268	CHEMBL3338	Enzyme	0	1/0
Endothelin-converting enzyme 1	ECE1	P42892	CHEMBL4791	Protease	0	6/0
Toll-like receptor (TLR7/TLR9)	TLR7	Q9NYK1	CHEMBL5936	Toll-like and Il-1 receptors	0	1/0
Glutathione *S*-transferase Mu 1	GSTM1	P09488	CHEMBL2081	Enzyme	0	1/0
Glutathione *S*-transferase A1	GSTA1	P08263	CHEMBL3409	Enzyme	0	2/0
Glyoxalase I	GLO1	Q04760	CHEMBL2424	Enzyme	0	4/0
2′-deoxynucleoside 5′-phosphate *N*-hydrolase 1	DNPH1	O43598	CHEMBL3351218	Hydrolase	0	1/0
Cysteinyl leukotriene receptor 1	CYSLTR1	Q9Y271	CHEMBL1798	Family A G protein-coupled receptor	0	1/0
Leukotriene A4 hydrolase	LTA4H	P09960	CHEMBL4618	Protease	0	3/0
GABA transporter 1 (by homology)	SLC6A1	P30531	CHEMBL1903	Electrochemical transporter	0	2/0
Serine/threonine-protein kinase PIM1	PIM1	P11309	CHEMBL2147	Kinase	0	4/0
Serine/threonine-protein kinase PIM2	PIM2	Q9P1W9	CHEMBL4523	Kinase	0	3/0
Serine/threonine-protein kinase PIM3	PIM3	Q86V86	CHEMBL5407	Kinase	0	3/0
Tyrosine-protein kinase SRC	SRC	P12931	CHEMBL267	Kinase	0	3/0
Folylpoly-gamma-glutamate synthetase	FPGS	Q05932	CHEMBL3171	Enzyme	0	2/0
DNA (cytosine-5)-methyltransferase 3B	DNMT3B	Q9UBC3	CHEMBL6095	Reader	0	4/0
Cholesteryl ester transfer protein	CETP	P11597	CHEMBL3572	Other ion channel	0	0/1
Dipeptidyl peptidase I	CTSC	P53634	CHEMBL2252	Protease	0	9/0
Tyrosine-protein kinase ZAP-70	ZAP70	P43403	CHEMBL2803	Kinase	0	1/0
Histone-lysine *N*-methyltransferase, H3 lysine-79 specific	DOT1L	Q8TEK3	CHEMBL1795117	Writer	0	4/0
Glutathione *S*-transferase Pi	GSTP1	P09211	CHEMBL3902	Enzyme	0	1/0
Lysine-specific demethylase 4A	KDM4A	O75164	CHEMBL5896	Eraser	0	1/0
Acyl coenzyme A:cholesterol acyltransferase 1	SOAT1	P35610	CHEMBL2782	Enzyme	0	3/0
Diacylglycerol *O*-acyltransferase 1	DGAT1	O75907	CHEMBL6009	Enzyme	0	3/0
Cathepsin K	CTSK	P43235	CHEMBL268	Protease	0	1/0
Glutamate receptor ionotropic kainate 2	GRIK2	Q13002	CHEMBL3683	Ligand-gated ion channel	0	1/0
Lysine-specific demethylase 5A	KDM5A	P29375	CHEMBL2424504	Eraser	0	1/0
Dihydrofolate reductase	DHFR	P00374	CHEMBL202	Oxidoreductase	0	2/0
Nitric-oxide synthase, brain	NOS1	P29475	CHEMBL3568	Enzyme	0	1/0
Receptor protein-tyrosine kinase erbB-2	ERBB2	P04626	CHEMBL1824	Kinase	0	1/0
Adenosine A2a receptor	ADORA2A	P29274	CHEMBL251	Family A G protein-coupled receptor	0	1/0
Prostanoid EP1 receptor	PTGER1	P34995	CHEMBL1811	Family A G protein-coupled receptor	0	1/0
Prostanoid FP receptor	PTGFR	P43088	CHEMBL1987	Family A G protein-coupled receptor	0	1/0
Tryptophan 5-hydroxylase 1	TPH1	P17752	CHEMBL5689	Enzyme	0	1/0
Protein farnesyltransferase	FNTA FNTB	P49354 P49356	CHEMBL2094108	Enzyme	0	0/10
Glutamate receptor ionotropic kainate 1	GRIK1	P39086	CHEMBL1918	Ligand-gated ion channel	0	0/1
Muscarinic acetylcholine receptor M4	CHRM4	P08173	CHEMBL1821	Family A G protein-coupled receptor	0	0/1
Glucocorticoid receptor	NR3C1	P04150	CHEMBL2034	Nuclear receptor	0	0/1
Muscarinic acetylcholine receptor M5	CHRM5	P08912	CHEMBL2035	Family A G protein-coupled receptor	0	0/1
Muscarinic acetylcholine receptor M1	CHRM1	P11229	CHEMBL216	Family A G protein-coupled receptor	0	0/1
Histamine H1 receptor	HRH1	P35367	CHEMBL231	Family A G protein-coupled receptor	0	0/1
Muscarinic acetylcholine receptor M3	CHRM3	P20309	CHEMBL245	Family A G protein-coupled receptor	0	0/1
Serine/threonine-protein kinase AKT	AKT1	P31749	CHEMBL4282	Kinase	0	0/1
Geranylgeranyl pyrophosphate synthetase	GGPS1	O95749	CHEMBL4769	Enzyme	0	9/4
Lysophosphatidic acid receptor 6	LPAR6	P43657	CHEMBL2331058	Family A G protein-coupled receptor	0	2/1
Lysophosphatidic acid receptor Edg-4	LPAR2	Q9HBW0	CHEMBL3724	Family A G protein-coupled receptor	0	3/1
Lysophosphatidic acid receptor 5	LPAR5	Q9H1C0	CHEMBL5700	Family A G protein-coupled receptor	0	2/1
Lysophosphatidic acid receptor 4	LPAR4	Q99677	CHEMBL5968	Family A G protein-coupled receptor	0	2/1
Autotaxin	ENPP2	Q13822	CHEMBL3691	Enzyme	0	8/1
Vanilloid receptor	TRPV1	Q8NER1	CHEMBL4794	Voltage-gated ion channel	0	1/1
Putative P2Y purinoceptor 10	P2RY10	O00398	CHEMBL3562166	Family A G protein-coupled receptor	0	1/15
Probable G-protein coupled receptor 34	GPR34	Q9UPC5	CHEMBL3562165	Family A G protein-coupled receptor	0	1/6
Probable G-protein coupled receptor 174	GPR174	Q9BXC1	CHEMBL3562167	Family A G protein-coupled receptor	0	1/7

**TABLE 1B:** SwissTargePrediction of the molecular targets of S1P (Isomeric SMILES) in Homo sapiens.

**Target**	**Common name**	**Uniprot ID**	**ChEMBL ID**	**Target class**	**Probability**	**Known actives (3D/2D)**
Sphingosine 1-phosphate receptor Edg-8	S1PR5	Q9H228	CHEMBL2274	Family A G protein-coupled receptor	1	48/1
Sphingosine 1-phosphate receptor Edg-5	S1PR2	O95136	CHEMBL2955	Family A G protein-coupled receptor	1	10/1
Sphingosine 1-phosphate receptor Edg-6	S1PR4	O95977	CHEMBL3230	Family A G protein-coupled receptor	1	42/1
Sphingosine 1-phosphate receptor Edg-3	S1PR3	Q99500	CHEMBL3892	Family A G protein-coupled receptor	1	99/2
Sphingosine 1-phosphate receptor Edg-1	S1PR1	P21453	CHEMBL4333	Family A G protein-coupled receptor	1	141/2
Lysophosphatidic acid receptor Edg-7	LPAR3	Q9UBY5	CHEMBL3250	Family A G protein-coupled receptor	0.097239989	4/10
Endothelin-converting enzyme 1	ECE1	P42892	CHEMBL4791	Protease	0	7/0
Sphingosine kinase 2	SPHK2	Q9NRA0	CHEMBL3023	Enzyme	0	0/2
Sphingosine kinase 1	SPHK1	Q9NYA1	CHEMBL4394	Enzyme	0	0/3
Lysophosphatidic acid receptor Edg-2	LPAR1	Q92633	CHEMBL3819	Family A G protein-coupled receptor	0	3/5
Farnesyl diphosphate synthase	FDPS	P14324	CHEMBL1782	Transferase	0	11/0
Glyoxalase I	GLO1	Q04760	CHEMBL2424	Enzyme	0	6/0
NAD-dependent deacetylase sirtuin 1	SIRT1	Q96EB6	CHEMBL4506	Eraser	0	4/0
Cysteinyl leukotriene receptor 1	CYSLTR1	Q9Y271	CHEMBL1798	Family A G protein-coupled receptor	0	1/0
Glutathione *S*-transferase Mu 1	GSTM1	P09488	CHEMBL2081	Enzyme	0	2/0
Angiotensin-converting enzyme	ACE	P12821	CHEMBL1808	Protease	0	7/0
Neprilysin	MME	P08473	CHEMBL1944	Protease	0	8/0
Indoleamine 2,3-dioxygenase	IDO1	P14902	CHEMBL4685	Enzyme	0	3/0
Disks large homolog 4	DLG4	P78352	CHEMBL5666	Unclassified protein	0	4/0
DNA (cytosine-5)-methyltransferase 3B	DNMT3B	Q9UBC3	CHEMBL6095	Reader	0	4/0
Glutathione *S*-transferase A1	GSTA1	P08263	CHEMBL3409	Enzyme	0	2/0
Carbonic anhydrase II	CA2	P00918	CHEMBL205	Lyase	0	22/0
Carbonic anhydrase I	CA1	P00915	CHEMBL261	Lyase	0	20/0
Carbonic anhydrase XII	CA12	O43570	CHEMBL3242	Lyase	0	6/0
Carbonic anhydrase IX	CA9	Q16790	CHEMBL3594	Lyase	0	6/0
Squalene synthetase (by homology)	FDFT1	P37268	CHEMBL3338	Enzyme	0	6/0
Thrombin and coagulation factor X	F10	P00742	CHEMBL244	Protease	0	3/0
Dipeptidyl peptidase I	CTSC	P53634	CHEMBL2252	Protease	0	7/0
Glutamate receptor ionotropic kainate 2	GRIK2	Q13002	CHEMBL3683	Ligand-gated ion channel	0	2/0
Serine/threonine-protein kinase PIM1	PIM1	P11309	CHEMBL2147	Kinase	0	4/0
Glutathione *S*-transferase Pi	GSTP1	P09211	CHEMBL3902	Enzyme	0	3/0
Serine/threonine-protein kinase PIM2	PIM2	Q9P1W9	CHEMBL4523	Kinase	0	3/0
Serine/threonine-protein kinase PIM3	PIM3	Q86V86	CHEMBL5407	Kinase	0	3/0
Leukotriene A4 hydrolase	LTA4H	P09960	CHEMBL4618	Protease	0	7/0
Cholesteryl ester transfer protein	CETP	P11597	CHEMBL3572	Other ion channel	0	0/1
Caspase-1	CASP1	P29466	CHEMBL4801	Protease	0	2/0
Tryptophan 5-hydroxylase 1	TPH1	P17752	CHEMBL5689	Enzyme	0	1/0
Histone-lysine *N*-methyltransferase, H3 lysine-79 specific	DOT1L	Q8TEK3	CHEMBL1795117	Writer	0	2/0
Calcium sensing receptor	CASR	P41180	CHEMBL1878	Family C G protein-coupled receptor	0	3/0
EZH2/SUZ12/EED/RBBP7/RBBP4	EZH2	Q15910	CHEMBL2189110	Writer	0	1/0
2′-deoxynucleoside 5′-phosphate *N*-hydrolase 1	DNPH1	O43598	CHEMBL3351218	Hydrolase	0	2/0
Transmembrane protease serine 11D	TMPRSS11D	O60235	CHEMBL1795138	Protease	0	1/0
Thrombin	F2	P00734	CHEMBL204	Protease	0	1/0
Glycogen synthase kinase-3 beta	GSK3B	P49841	CHEMBL262	Kinase	0	1/0
Matriptase	ST14	Q9Y5Y6	CHEMBL3018	Protease	0	1/0
Matrix metalloproteinase 2	MMP2	P08253	CHEMBL333	Protease	0	1/0
Matrix metalloproteinase 12	MMP12	P39900	CHEMBL4393	Protease	0	1/0
Matrix metalloproteinase 8	MMP8	P22894	CHEMBL4588	Protease	0	1/0
Glutamate receptor ionotropic kainate 3	GRIK3	Q13003	CHEMBL3684	Ligand-gated ion channel	0	1/0
GABA transporter 1 (by homology)	SLC6A1	P30531	CHEMBL1903	Electrochemical transporter	0	2/0
Integrin alpha-V/beta-3	ITGAV ITGB3	P06756 P05106	CHEMBL1907598	Membrane receptor	0	1/0
Cathepsin K	CTSK	P43235	CHEMBL268	Protease	0	1/0
Tyrosine-protein kinase ZAP-70	ZAP70	P43403	CHEMBL2803	Kinase	0	1/0
Caspase-3	CASP3	P42574	CHEMBL2334	Protease	0	1/0
Aminopeptidase N	ANPEP	P15144	CHEMBL1907	Protease	0	1/0
Integrin alpha-5/beta-1	ITGB1 ITGA5	P05556 P08648	CHEMBL2095226	Membrane receptor	0	1/0
C3a anaphylatoxin chemotactic receptor	C3AR1	Q16581	CHEMBL4761	Family A G protein-coupled receptor	0	1/0
Endoplasmic reticulum aminopeptidase 2	ERAP2	Q6P179	CHEMBL5043	Protease	0	1/0
Endoplasmic reticulum aminopeptidase 1	ERAP1	Q9NZ08	CHEMBL5939	Protease	0	1/0
Prostanoid EP4 receptor (by homology)	PTGER4	P35408	CHEMBL1836	Family A G protein-coupled receptor	0	1/0
Protein farnesyltransferase	FNTA FNTB	P49354 P49356	CHEMBL2094108	Enzyme	0	0/10
Muscarinic acetylcholine receptor M4	CHRM4	P08173	CHEMBL1821	Family A G protein-coupled receptor	0	0/1
Glucocorticoid receptor	NR3C1	P04150	CHEMBL2034	Nuclear receptor	0	0/1
Muscarinic acetylcholine receptor M5	CHRM5	P08912	CHEMBL2035	Family A G protein-coupled receptor	0	0/1
Muscarinic acetylcholine receptor M1	CHRM1	P11229	CHEMBL216	Family A G protein-coupled receptor	0	0/1
Histamine H1 receptor	HRH1	P35367	CHEMBL231	Family A G protein-coupled receptor	0	0/1
Muscarinic acetylcholine receptor M3	CHRM3	P20309	CHEMBL245	Family A G protein-coupled receptor	0	0/1
Serine/threonine-protein kinase AKT	AKT1	P31749	CHEMBL4282	Kinase	0	0/1
Geranylgeranyl pyrophosphate synthetase	GGPS1	O95749	CHEMBL4769	Enzyme	0	12/4
Lysophosphatidic acid receptor 6	LPAR6	P43657	CHEMBL2331058	Family A G protein-coupled receptor	0	2/1
Lysophosphatidic acid receptor Edg-4	LPAR2	Q9HBW0	CHEMBL3724	Family A G protein-coupled receptor	0	3/1
Lysophosphatidic acid receptor 5	LPAR5	Q9H1C0	CHEMBL5700	Family A G protein-coupled receptor	0	2/1
Lysophosphatidic acid receptor 4	LPAR4	Q99677	CHEMBL5968	Family A G protein-coupled receptor	0	2/1
Putative P2Y purinoceptor 10	P2RY10	O00398	CHEMBL3562166	Family A G protein-coupled receptor	0	3/15
Probable G-protein coupled receptor 34	GPR34	Q9UPC5	CHEMBL3562165	Family A G protein-coupled receptor	0	3/6
Autotaxin	ENPP2	Q13822	CHEMBL3691	Enzyme	0	5/1
Vanilloid receptor	TRPV1	Q8NER1	CHEMBL4794	Voltage-gated ion channel	0	1/1
Probable G-protein coupled receptor 174	GPR174	Q9BXC1	CHEMBL3562167	Family A G protein-coupled receptor	0	2/7
Glutamate receptor ionotropic kainate 1	GRIK1	P39086	CHEMBL1918	Ligand-gated ion channel	0	1/1
Enteropeptidase	TMPRSS15	P98073	CHEMBL1741195	Protease	0	2/0
Trypsin I	PRSS1	P07477	CHEMBL209	Protease	0	2/0
Vascular endothelial growth factor receptor 2	KDR	P35968	CHEMBL279	Kinase	0	1/0
Carbonic anhydrase IV	CA4	P22748	CHEMBL3729	Lyase	0	10/0
Thymidylate synthase (by homology)	TYMS	P04818	CHEMBL1952	Transferase	0	3/0
Acyl coenzyme A:cholesterol acyltransferase 1	SOAT1	P35610	CHEMBL2782	Enzyme	0	4/0
Diacylglycerol *O*-acyltransferase 1	DGAT1	O75907	CHEMBL6009	Enzyme	0	5/0
Metastin receptor	KISS1R	Q969F8	CHEMBL5413	Family A G protein-coupled receptor	0	2/0
Dihydrofolate reductase	DHFR	P00374	CHEMBL202	Oxidoreductase	0	1/0
Integrin alpha-IIb/beta-3	ITGA2B ITGB3	P08514 P05106	CHEMBL2093869	Membrane receptor	0	1/0
Growth factor receptor-bound protein 2	GRB2	P62993	CHEMBL3663	Other cytosolic protein	0	1/0
MAP kinase ERK2 (by homology)	MAPK1	P28482	CHEMBL4040	Kinase	0	1/0
MAP kinase p38 alpha (by homology)	MAPK14	Q16539	CHEMBL260	Kinase	0	1/0
Folylpoly-gamma-glutamate synthetase (by homology)	FPGS	Q05932	CHEMBL3171	Enzyme	0	1/0

### Over Representation Analysis (ORA) of the Molecular Targets of S1P Using WebGestalt

**FIGURE 2:**
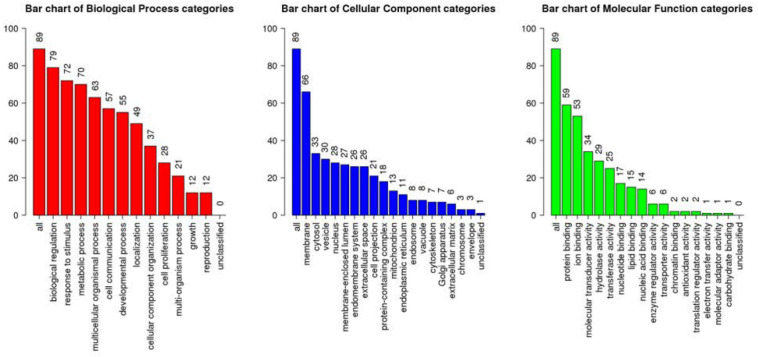
Over Representation Analysis (ORA) using Web-based Gene Set Analysis Toolkit (WebGestalt). GO Slim Summary for S1P molecular targets in humans showing Biological Process, Cellular Component, and Molecular Function categories in the red, blue, and green bar, respectively. The height of the bar represents the number of IDs in the user list and also in the GO category.

All the 93 molecular targets of S1P obtained using isomeric SMILES were used as input molecules in WebGestalt Open Source Tool to perform the ORA. GO Slim Summary for S1P Molecular Targets in Humans showing Biological Process, Cellular Component, and Molecular Function category in the red, blue, and green bar, respectively. The height of the bar represents the number of IDs in the user list and also in the category (Figure 2). The query list had 93 targets of S1P in which 89 were mapped to 89 unique Entrez gene IDs unambiguously, and the remaining 4 could not be mapped to any Entrez gene ID. Therefore, the GO Slim summary was established upon the 89 distinctive Entrez gene IDs (Figure 2). The reference list was mapped to 61506 Entrez gene IDs and 16671 IDs were annotated to the selected functional categories that were used as the reference for the enrichment analysis. The GO Biological Processes such as sphingolipid mediated signaling pathway, S1P receptor signaling pathway, chemical homeostasis, positive regulation of cytosolic calcium ion concentration, and cellular calcium ion homeostasis, and phospholipase C-activating G protein-coupled receptor signaling pathway were significantly regulated (*P* < 0.01; *Q* < 0.01) in ORA (Table 2). The GO Molecular Functions such as the bioactive lipid receptor activity, S1P receptor activity, transmembrane signaling receptor activity, molecular transducer activity, G protein-coupled receptor activity, etc. were significantly regulated (*P* < 0.01; *Q* < 0.01) in the ORA (Table 3). The molecules involved in the bioactive receptor activity (GO:0045125) comprise of GPR174, LPAR1, LPAR2, LPAR3, LPAR4, S1PR1, S1PR2, S1PR3, S1PR4, S1PR5, SPHK1, and SPHK2 (Table 4).

**TABLE 2:** The biological processes regulated by the molecular targets of S1P in Homo sapiens.

**Gene set**	**Description**	**Size**	**Expect**	**Ratio**	***P*-value**	**FDR**
GO:0003376	Sphingosine-1-phosphate receptor signaling pathway	10	0.053402	131.08	1.1546e-14	1.0497e-10
GO:0090520	Sphingolipid mediated signaling pathway	11	0.058742	119.16	3.1530e-14	1.4332e-10
GO:0007200	Phospholipase C-activating G protein-coupled receptor signaling pathway	96	0.51266	23.407	1.0836e-13	3.2836e-10
GO:0050801	Ion homeostasis	776	4.1440	6.0328	1.6986e-13	3.8606e-10
GO:0007186	G protein-coupled receptor signaling pathway	1290	6.8889	4.5000	2.6357e-13	4.5957e-10
GO:0048878	Chemical homeostasis	1119	5.9757	4.8530	3.0331e-13	4.5957e-10
GO:0051482	Positive regulation of cytosolic calcium ion concentration involved in phospholipase C-activating G protein-coupled signaling pathway	31	0.16555	48.325	3.4222e-12	4.4444e-9
GO:0007204	Positive regulation of cytosolic calcium ion concentration	301	1.6074	9.9539	4.6408e-12	5.2737e-9
GO:0006874	Cellular calcium ion homeostasis	439	2.3444	7.6780	1.4333e-11	1.4478e-8

**TABLE 3:** The molecular processes regulated by the molecular targets of S1P in Homo sapiens.

**Gene set**	**Description**	**Size**	**Expect**	**Ratio**	***P*-value**	**FDR**
GO:0045125	Bioactive lipid receptor activity	14	0.074741	160.56	0	0
GO:0038036	Sphingosine-1-phosphate receptor activity	8	0.042709	163.90	7.7716e-16	7.2936e-13
GO:0004930	G protein-coupled receptor activity	806	4.3029	6.2748	5.4401e-15	3.4037e-12
GO:0038023	Signaling receptor activity	1429	7.6289	4.4568	1.5210e-14	7.1373e-12
GO:0004888	Transmembrane signaling receptor activity	1211	6.4651	4.7950	4.7962e-14	1.5594e-11
GO:0060089	Molecular transducer activity	1488	7.9439	4.2800	4.9849e-14	1.5594e-11
GO:0004175	Endopeptidase activity	436	2.3276	7.7332	1.2733e-11	3.4142e-9
GO:0070011	Peptidase activity, acting on L-amino acid peptides	610	3.2566	6.1415	5.0832e-11	1.1927e-8
GO:0008233	Peptidase activity	634	3.3847	5.9090	1.0096e-10	2.1055e-8
GO:0008236	Serine-type peptidase activity	204	1.0891	11.019	8.4807e-10	1.5918e-7

**TABLE 4:** The genes involved in the bioactive receptor activity (GO:0045125) regulated by S1P in Homo sapiens.

**Gene symbol**	**Gene Name**	**Entrez Gene ID**
GPR174	G protein-coupled receptor 174	84636
LPAR1	Lysophosphatidic acid receptor 1	1902
LPAR2	Lysophosphatidic acid receptor 2	9170
LPAR3	Lysophosphatidic acid receptor 3	23566
LPAR4	Lysophosphatidic acid receptor 4	2846
S1PR1	Sphingosine-1-phosphate receptor 1	1901
S1PR2	Sphingosine-1-phosphate receptor 2	9294
S1PR3	Sphingosine-1-phosphate receptor 3	1903
S1PR4	Sphingosine-1-phosphate receptor 4	8698
S1PR5	Sphingosine-1-phosphate receptor 5	53637
SPHK1	Sphingosine kinase 1	8877
SPHK2	sphingosine kinase 2	56848

### Identification of S1P-Induced Molecular Targets in Asthma and Other Respiratory Diseases

Then, we used the Open Targets Platform to find the S1P molecular targets associated with respiratory diseases. Our findings showed that about 109 types of respiratory diseases were significantly (*P* < 0.05) affected by the molecular targets of S1P. The top 15 respiratory diseases significantly (*P* < 0.01; *Q* < 0.01) induced by S1P and its molecular targets were lung disease, respiratory system disease, respiratory system neoplasm, bronchial disease, lung carcinoma, asthma, interstitial lung disease, COPD, Rare genetic respiratory disease, idiopathic pulmonary fibrosis, pulmonary fibrosis, acute lung injury, pneumonia, whooping cough, and non-small cell lung adenocarcinoma (Table 5).

**TABLE 5:** Top 15 respiratory diseases associated with the molecular targets of S1P.

**Disease full name**	**Relevance (*p*-value)**	**Number of associated targets**	**Therapeutic area**	**Highest associated targets (max 10)**
Lung disease	6.00E-42	82	Respiratory system disease	KDR TYMS CHRM3 MMP8 NR3C1 CHRM1 AKT1 CA4 ACE MMP2…
Respiratory system disease	2.00E-39	84	Respiratory system disease	KDR TYMS CHRM3 MMP8 HRH1 NR3C1 CHRM1 AKT1 CA4 MMP2…
Respiratory system neoplasm	2.00E-38	77	Neoplasm, respiratory system disease	KDR TYMS NR3C1 AKT1 MMP2 DHFR FDPS GRB2 MMP8 MMP12…
Bronchial disease	2.00E-38	58	Respiratory system disease	NR3C1 CYSLTR1 HRH1 CHRM1 CHRM3 DHFR GSTM1 MAPK14 CASP1 S1PR2…
Lung carcinoma	5.00E-38	78	Neoplasm, respiratory system disease	KDR TYMS NR3C1 AKT1 MMP2 DHFR FDPS GRB2 MMP12 IDO1…
Asthma	1.00E-35	55	Respiratory system disease	NR3C1 CYSLTR1 HRH1 CHRM3 CHRM1 DHFR GSTM1 MAPK14 CASP1 S1PR2…
Interstitial lung disease	3.00E-32	43	Respiratory system disease	NR3C1 KDR MMP8 CTSK EZH2 SOAT1 CYSLTR1 SIRT1 CASP3 MMP12…
Chronic obstructive pulmonary disease	8.00E-32	48	Respiratory system disease	CHRM3 MMP8 ACE NR3C1 CA1 CHRM1 CA12 CA4 CA2 MMP12…
Rare genetic respiratory disease	3.00E-31	44	Genetic disorder, respiratory system disease	MMP8 FDPS NR3C1 CHRM3 SIRT1 PRSS1 EZH2 CTSK HRH1 KISS1R…
Idiopathic pulmonary fibrosis	2.00E-29	39	Neoplasm, respiratory system disease	NR3C1 KDR MMP8 CTSK EZH2 SOAT1 CASP3 MMP2 ANPEP TYMS…
Pulmonary fibrosis	7.00E-29	37	Neoplasm, respiratory system disease	NR3C1 KDR MMP8 CTSK EZH2 SOAT1 CASP3 MMP12 ANPEP MMP2…
Acute lung injury	9.00E-28	32	Respiratory system disease	MAPK14 MMP8 MMP2 GSK3B IDO1 S1PR1 SIRT1 CASP1 CASP3 SOAT1…
Pneumonia	2.00E-27	37	Infectious disease, respiratory system disease	MMP8 NR3C1 CHRM3 CHRM1 MMP12 ACE DHFR SOAT1 F2 MMP2…
Whooping cough	3.00E-27	32	Infectious disease, respiratory system disease	CASP1 CASP3 AKT1 CA1 S1PR4 S1PR1 MAPK1 HRH1 MMP2 TRPV1…
Non-small cell lung adenocarcinoma	3.00E-26	42	Neoplasm, respiratory system disease	SIRT1 EZH2 CASR GSK3B TYMS MMP2 IDO1 KDR CASP3 CA9…

### Ingenuity Pathway Analysis of the Differentially Regulated Gene Networks by S1P/S1PR Axis in Respiratory Diseases

**FIGURE 3:**
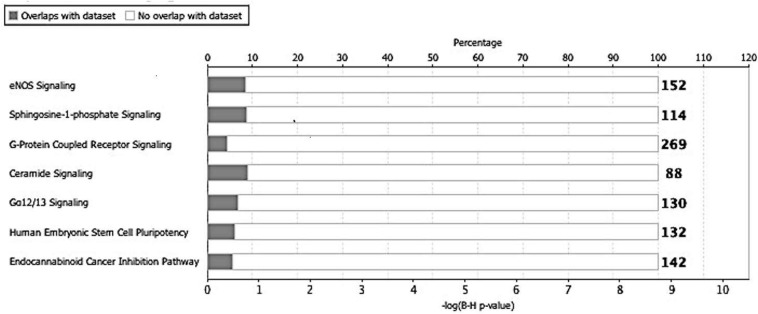
Canonical pathways regulated by the molecular targets of S1P. The IPA core analysis of the molecular targets of S1P revealed that the canonical pathways such as eNOS signaling, sphingosine-1-phosphate signaling, G-protein coupled receptor signaling, ceramide signaling, G a12/13 signaling, human embryonic stem cell pluripotency, and endocannabinoid cancer inhibition pathway were potentially regulated (*P* < 0.05).

Next, we utilized the IPA to deduce the canonical pathways, upstream regulators, causal functions, diseases, and bio functions, and non-directional unique networks significantly impacted by the S1P mediated signaling molecules. The IPA core analysis of the molecular targets of S1P revealed that the canonical pathways such as eNOS signaling, S1P signaling, GPCRs signaling, ceramide signaling, G a12/13 signaling, human embryonic stem cell pluripotency, and endocannabinoid cancer inhibition pathway (Figure 3) were potentially regulated (*P* < 0.05). About 263 molecules play a significant role (*P* < 0.05) as upstream regulators of the molecular targets of S1P (Supplementary Table S1). Furthermore, we have filtered the upstream regulators based on the molecule types such as cytokines, and growth factors using the IPA. The cytokines such as Interleukin-13 (IL-13), Interferon-gamma (IFN-γ), IFN-β1, Tumor Necrosis Factor-alpha (TNF-α), Interleukin-4 (IL-4), Interleukin-5 (IL-5), Interleukin-1 (IL-1β), Interleukin-21 (IL-21), Tissue Inhibitor of Metalloproteinases-1 (TIMP-1) that has cytokine-like activity, and cytokine-inducing Corticotropin-Releasing Hormone (CRH) were found to be the upstream regulators of the S1P signaling. The growth factors such as Hepatocyte Growth Factor (HGF), Vascular Endothelial Growth Factors (VEGF), Colony Stimulating Factor 1 (CSF1) or Macrophage Colony-Stimulating Factor (M-CSF), Colony Stimulating Factor-2 (CSF2) or Granulocyte-Macrophage Colony-Stimulating Factor (GM-CSF), VEGF-A, VEGF-B, VEGF-D, Fibroblast Growth Factor 16 (FGF16), Insulin-like Growth Factor (IGF), and Transforming Growth Factor-beta 1 (TGF-β1) were identified as the upstream regulators of S1P signaling (Table 6).

**TABLE 6:** Upstream regulators of the S1P molecular targets – Focus on cytokines and growth Factors.

**Upstream regulator**	**Molecule type**	***p*-value of overlap**	**Target molecules in dataset**
HGF	Growth factor	0.000000328	AKT1, CA9, CTSK, MAPK14, MMP2, MMP8, ST14
IL13	Cytokine	0.0000174	C3AR1, CASP1, CTSC, CYSLTR1, ENPP2, LTA4H, ST14
IFNG	Cytokine	0.000145	ACE, CASP1, CASP3, ERAP2, IDO1, MMP2, PIM1, SOAT1
IFNB1	Cytokine	0.000528	CASP1, CASP3, IDO1
VEGFA	Growth factor	0.000775	KDR, MMP12, MMP2
TNF	Cytokine	0.00118	CTSC, GSTA1, IDO1, MMP12, MMP2, MMP8, NR3C1, SOAT1
IL4	Cytokine	0.00132	CYSLTR1, IDO1, PIM1, ST14
VEGFB	Growth factor	0.0044	MMP12
CSF1	Growth factor	0.00767	CTSK, MMP12
LIF	Cytokine	0.0131	ERAP1
CSF2	Growth factor	0.0133	CASP3, CTSC, PIM1
CRH	Cytokine	0.0175	TPH1
FGF16	Growth factor	0.0175	MMP2
VEGFD	Growth factor	0.0175	MMP12
TGFB1	Growth factor	0.0195	KDR, MAPK1, MMP12, MMP2, SPHK1
IL5	Cytokine	0.0197	CTSC, PIM1
IL1B	Cytokine	0.0236	GSTA1, KDR, MMP12, MMP8
IL21	Cytokine	0.0296	IDO1, MMP2
TIMP1	Cytokine	0.0389	MME
IGF2	Growth factor	0.0389	MMP12

### Validation of Biomarkers in Asthma Using Luminex xMAP Technology

**FIGURE 4:**
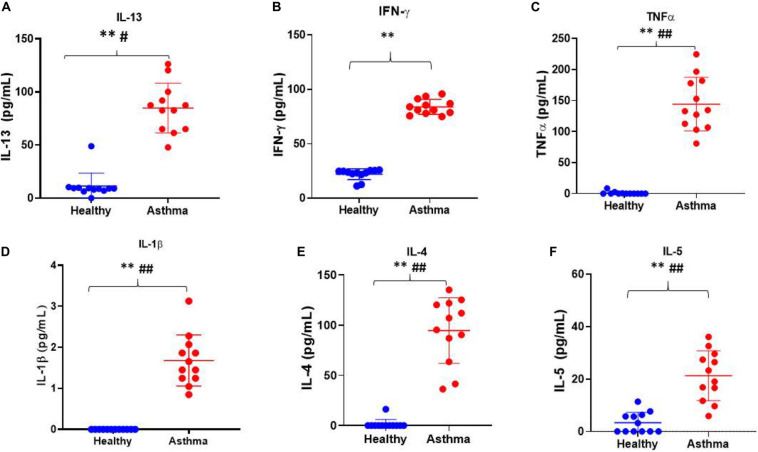
Luminex xMAP assay to validate the cytokines that were upstream of the S1P signaling in asthma. The upstream regulator cytokines of S1P signaling such as IL-13 **(A)**, IFN-γ **(B)**, TNF-α **(C)**, IL-1β **(D)**, IL-4 **(E)**, and IL-5 **(F)** were significantly (*T*-test ^∗∗^*P* < 0.01, *F*-test ^#^*P* < 0.05, and ^##^*P* < 0.01) elevated in the serum of asthma patients compared the healthy controls.

**FIGURE 5:**
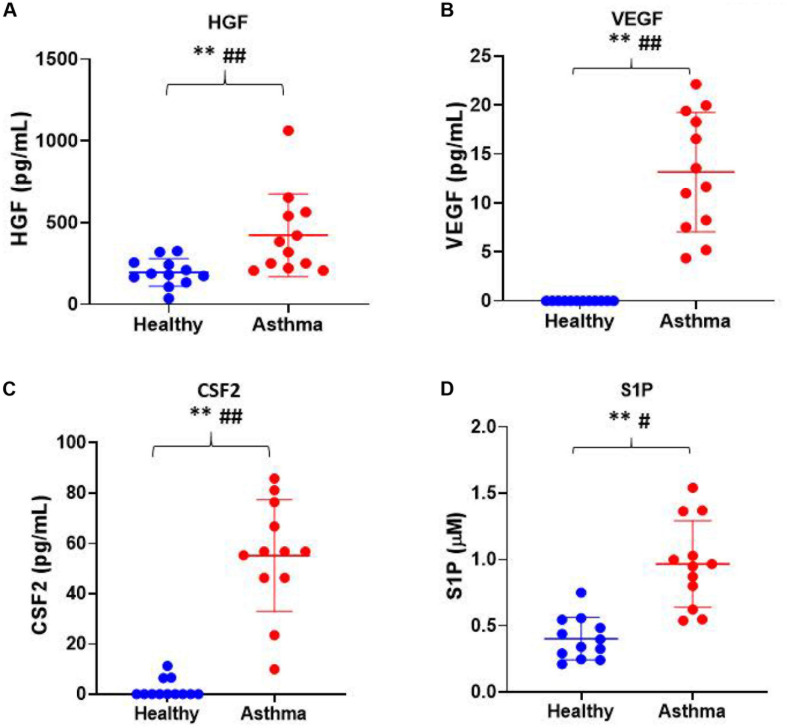
Luminex xMAP assay to validate the growth factors that were upstream of the S1P signaling in asthma. The growth factors that were found to be upstream of S1P signaling such as **(A)** HGF, **(B)** VEGF, **(C)** CSF2, and **(D)** S1P were significantly (*T*-test ^∗∗^*P* < 0.01, *F*-test ^#^*P* < 0.05, and ^##^*P* < 0.01) elevated in the serum of asthma patients compared to the healthy controls.

Besides, we have used the Luminex xMAP assay to validate the cytokines and growth factors that were upstream of the S1P signaling in asthma. Here, we have analyzed the levels of cytokines such as IL-13, IFN-γ, TNF-α, IL-4, IL-5, and IL-1β (Figure 4) and growth factors such as HGF, VEGF, and CSF2 or GM-CSF along with the estimation of serum S1P concentration in asthma patients compared with the healthy controls (Figure 5). The clinical and laboratory characteristics of asthma patients and healthy controls were given in Table 7. The upstream regulator cytokines of S1P signaling such as IL-13, IFN-γ, TNF-α, IL-4, IL-5, and IL-1β were significantly (*P* < 0.01) increased in the serum of asthma patients compared the healthy controls. Similarly, the growth factors that were found to be upstream of S1P signaling such as HGF, VEGF, and CSF2 were significantly (*P* < 0.01) increased in the serum of asthma patients compared to the healthy controls. The S1P levels were significantly increased in the serum of asthma patients compared to the healthy controls. All the cytokines and growth factors analyzed by the Luminex xMAP assay were positively correlated with the S1P levels in the serum of asthmatics (Supplementary Table S2).

**TABLE 7:** Clinical and laboratory parameters in healthy control and asthma patients.

**Clinical Parameters**	**Healthy (*n* = 12)**	**Asthma (*n* = 12)**
Age (Years)	34 (23 – 48)	33 (22 – 45)
Disease Duration (Years)	NA	8.5 (5 – 10)
Serum IgE (IU/L)	22 (14 – 65)	365 (79 – 621)
WBC Count (10^9^/L)	6.75 (5.2 – 7.3)	7.1 (5.7 – 8.1)
Eosinophils (percentage)	0.14 (0.11 – 0.19)	5.5 (3 – 10)
FEV1(Liter)	NA	2.3 (1.9 – 2.9)

*NA, either data not available or not applicable. The table shows data as medians with the range indicated in the parentheses.*

## Discussion

In the present study, to decode the biological and molecular functions of bioactive lipid molecule, S1P in the development of asthma and other respiratory diseases, we have applied both next-generation knowledge discovery platforms such as SwissTargetPrediction, WebGestalt, Open Targets Platform, and IPA and validated the key upstream regulators of S1P signaling using Luminex xMAP technology. Currently, knowledge discovery and big data analytical platforms are swiftly transforming the Biomedical Research landscape (Cirillo and Valencia, 2019; Paczkowska et al., 2020). All the chemical or biochemical compounds with pharmacological or pathological actions influencing diagnosis, treatment, or recuperation, and prevention of a disease can be used in next-generation knowledge discovery platforms using a unique SMILES code (Weininger, 1988; Ratnawati et al., 2018). SMILES, postulated by David Weininger, is a chemical annotation method that symbolizes a simple molecule structure (Weininger, 1988; Ratnawati et al., 2018). Here, we have used the isomeric SMILES of S1P to deduce its downstream molecular targets using the SwissTargetPrediction server (Gfeller et al., 2013; Daina and Zoete, 2019; Daina et al., 2019).

Several studies have shown that S1P augments airway hyperactivity, bronchoconstriction, and airway remodeling in asthma and other related respiratory diseases (Roviezzo et al., 2010; Petrache and Berdyshev, 2016; Liu et al., 2018) and the S1P receptors are differentially expressed in the lymphoid tissues, dendritic cells, and the lung (Aarthi et al., 2011). S1P is an effective biologically active paracrine mediator that regulates various cellular functions such as apoptosis, cell growth, cell proliferation, immune regulation, etc. (Le Stunff et al., 2004; Aarthi et al., 2011). Here, we have further identified that biological processes such as S1PR signaling pathway, sphingolipid mediated signaling pathway, chemical homeostasis, positive regulation of cytosolic calcium ion concentration, and cellular calcium ion homeostasis were regulated by S1P. Importantly, the interaction of S1P with the five types of S1PRs and four types of LPARs on the plasma membrane triggers an intracellular cascade of reactions leading to the biosynthesis of various pro-inflammatory mediators that contribute to the pathogenicity in asthma. Also, the S1P regulates various molecular functions that are essential for the pathogenesis of asthma and respiratory diseases such as the bioactive lipid receptor activity, S1P receptor activity, G protein-coupled receptor activity, signaling receptor activity, transmembrane signaling receptor activity, molecular transducer activity, endopeptidase activity, peptidase activity acting on L-amino acid peptides, peptidase activity, and serine-type peptidase activity. We found that the S1P signaling was associated with more than 100 types of respiratory diseases such as lung disease, respiratory system disease, respiratory system neoplasm, bronchial disease, lung carcinoma, asthma, interstitial lung disease, COPD, rare genetic respiratory disease, idiopathic pulmonary fibrosis, pulmonary fibrosis, acute lung injury, pneumonia, whooping cough, non-small cell lung adenocarcinoma, etc., Besides, the S1P levels were found to be increased in the bronchoalveolar lavage fluids of allergic asthma patients and were also positively associated with augmented airway inflammation (Ammit et al., 2001; Kim, 2019). Altered sphingolipid metabolism according to the phenotype and genotype of asthma was deduced using metabolic studies in asthma patients (McGeachie et al., 2015; Kim, 2019). Similarly, the sphingolipid metabolism was found to be disturbed in aspirin-exacerbated respiratory disease (AERD), a serious type of adult-onset eosinophilic asthma (Trinh et al., 2016; Reinke et al., 2017).

In the present study, we have found several upstream regulators of S1P signaling including various cytokines such as IL-13, IFN-γ, IFN-β1, TNF-α, IL-4, IL-5, IL-1β, IL-21, TIMP-1, and CRH, and growth factors such as HGF, VEGF, CSF1, CSF2, VEGF-A, VEGF-B, VEGF-D, FGF16, IGF, and TGFβ1. Using multiplex xMAP technology, we have further validated that the levels of the upstream cytokines of S1P signaling such as IL-13, IFN-γ, TNF-α, IL-4, IL-5, and IL-1β were increased in the serum of asthma patients. Similarly, the growth factors such as HGF, VEGF, and CSF2 were increased in the serum of asthmatic patients. To further decode the association of the upstream cytokines and growth factors with S1P, we have estimated the S1P concentration in the serum of asthmatics. The levels of cytokines and growth factors analyzed using xMAP technology positively correlated with the S1P levels in the serum of asthmatics which further vouch for the significance of S1P signaling in the pathogenicity of asthma.

Asthma is a chronic disease and the incidence is ever-increasing around the globe (Al-Moamary et al., 2019). Most of the asthmatics have disease exacerbation caused by the Th2 type of immune cells compared to other types such as T-helper type 1 (Th1) and T-helper type 17 (Th17) and other immune cells such as mast cells, eosinophils, and bronchial smooth muscles, myofibroblasts and epithelial cells (Manikandan et al., 2012). The Th2 associated cytokines play a vital role in the development of allergy and airway remodeling in asthma (Komai-Koma et al., 2012). The asthmatics studied here had more eosinophils in their blood and the levels of Th2 serum cytokines such as IL-4, IL-5, and IL-13 were increased compared to the healthy controls. Here, most of the cytokines and growth factors examined were associated positively with an increase in the serum S1P. The sphingosine kinases regulate the expression of Th2, Th1, Th17, chemokines, and an array of other pro-inflammatory factors by catalyzing the conversion of sphingosine into S1P (Nayak et al., 2010; Aarthi et al., 2011). Many asthmatics have an uncontrolled disease state even though they are under a treatment regimen which could be due to the increased production of S1P (Aarthi et al., 2011) and a recent study found that ceramide/S1P rheostat was indeed dysregulated in uncontrolled asthma (Kim et al., 2020). Hence, it is prudent that the ceramide/S1P production may be targeted in controlling the airway inflammation in asthma (Kim et al., 2020). Importantly, the *in silico* results obtained using our next generation knowledge discovery pipeline coupled with xMAP assay also showed a potential association of S1P in asthma and other respiratory diseases which is detrimental for the patients due to its ability to trigger an array of cytokines, chemokines, and growth factors. More importantly, an increase in the upstream regulator Th2 cytokines such as IL-4, IL-5, and IL-13 is potentially harmful to asthmatics and play an underlying role in the disease pathogenesis. Similarly, the growth factors such as VEGF, CSF2, and HGF were found to be increased in asthmatics and may also be responsible for making the disease chronically worse. A recent study showed that the inhibition of S1P receptors potentially augments the inhibition of VEGF (Fischl et al., 2019). The S1P/S1PR signaling was regulated through the activation of SPHK1 and SPHK 2 to produce S1P by the phosphorylation of sphingosine (Nayak et al., 2010; Aarthi et al., 2011) and the airway hyperresponsiveness and inflammation was potentially reduced by SPHK inhibitors and FTY720, a synthetic analog of S1P and S1PR agonist (Aarthi et al., 2011) and other S1PR modulators (Marciniak et al., 2018), in animal models of allergic asthma (Sawicka et al., 2003; Idzko et al., 2006; Price et al., 2013).

We conclude that the S1P and its associated signaling molecules, either upstream or downstream, are pharmacological targets of great significance for the development of novel drugs for asthma and other related respiratory diseases in humans. However, one of the critical limitations of this study is the less number of samples used for the validation of biomarkers in the serum associated with S1P signaling in asthma. In future, we will be collecting more samples from patients suffering from atopic and non-atopic asthma and other respiratory diseases and disorders with varying severity that undergo different treatment regimens, for further validation of upstream regulator molecules associated with S1P signaling along with the key cytokines, chemokines, and growth factors that play a crucial role in the pathogenesis of an array of respiratory diseases and disorders.

## Data Availability Statement

The datasets generated for this study are available on request to the corresponding author.

## Ethics Statement

The studies involving human participants were reviewed and approved by our institutional ethical committee, and the study was accepted by the Scientific Research Committee, Deanship of Scientific Research (DSR), King Abdulaziz University (KAU), Jeddah. The patients/participants provided their written informed consent to participate in this study.

## Author Contributions

All authors designed and conducted the experiments, analyzed the data, wrote the manuscript, and contributed to the editing of the manuscript and the scientific discussions. SB, LD, and PP proposed the research idea.

## Conflict of Interest

The authors declare that the research was conducted in the absence of any commercial or financial relationships that could be construed as a potential conflict of interest.

The handling editor declared a past co-authorship with one of the authors PP.

## Footnotes

1 http://webgestalt.org/

## Supplementary Material

FIGURE S1 The flow diagram illustrates the essential steps in the *in silico* experiments using next-generation knowledge discovery platforms to uncover the molecular targets of S1P and their association with various respiratory diseases and disorders and the subsequent validation of disease-specific biomarkers and their relationship with S1P in Asthma.

TABLE S1 The list of 263 upstream regulators identified based on the IPA core analysis of the molecular targets of S1P.

TABLE S2 Simple linear regression analysis of S1P with upstream regulatory cytokines and growth factors in asthma patients.
