# Supplementary figures and images for: Decoding the Role of Sphingosine-1-Phosphate in Asthma and Other Respiratory System Diseases Using Next Generation Knowledge Discovery Platforms Coupled With Luminex Multiple Analyte Profiling Technology
Source: Front Cell Dev Biol. 2020 Jun 19;8:444. doi: 10.3389/fcell.2020.00444 (PMC7317666; doi:10.3389/fcell.2020.00444)

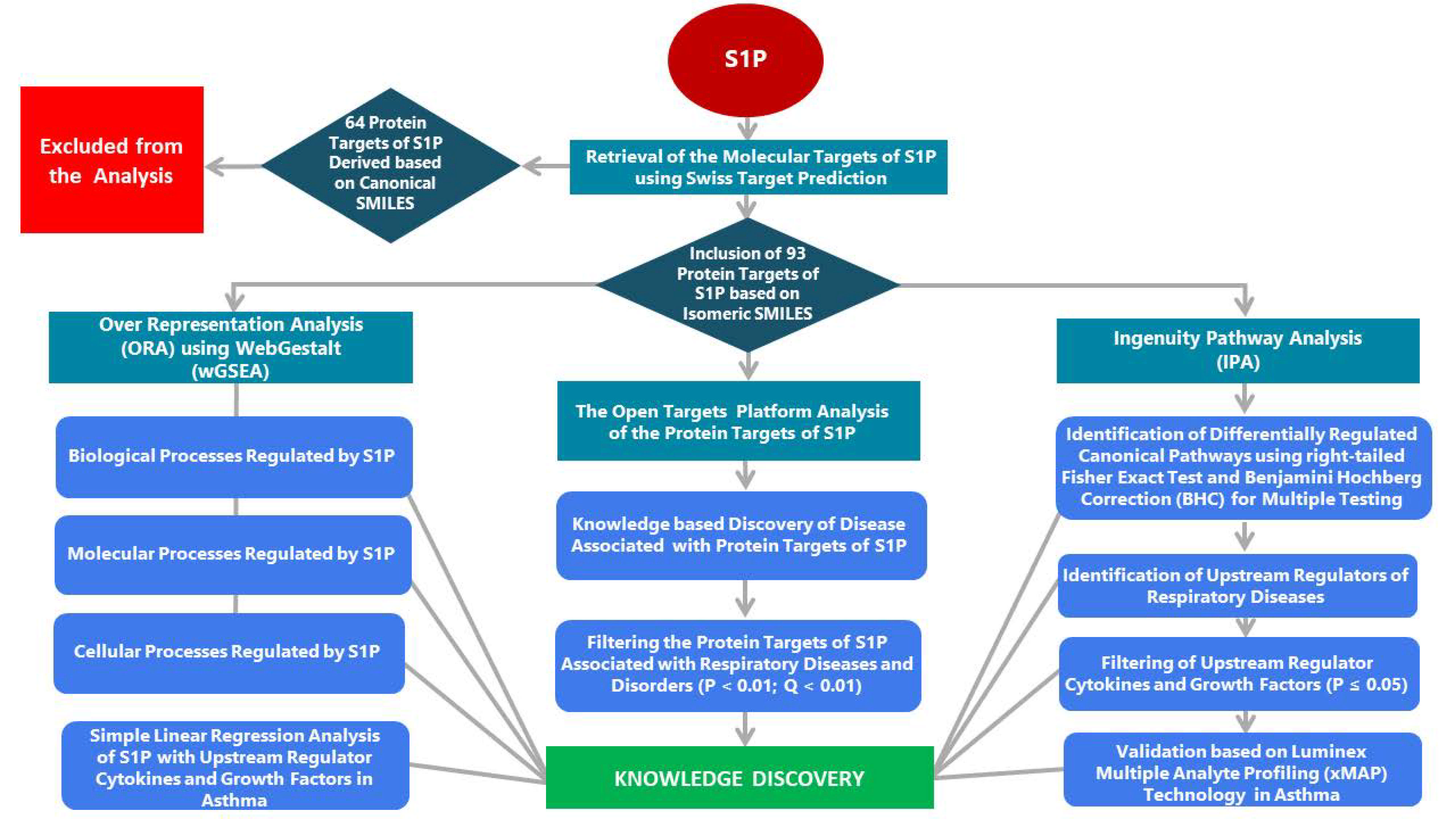

Supplement: FIGURE S1 — The flow diagram illustrates the essential steps in the in silico experiments using next-generation knowledge discovery platforms to uncover the molecular targets of S1P and their association with various respiratory diseases and disorders and the subsequent validation of disease-specific biomarkers and their relationship with S1P in Asthma. [file Image_1.tif]
