# Supplementary material for: Decoding the Role of Sphingosine-1-Phosphate in Asthma and Other Respiratory System Diseases Using Next Generation Knowledge Discovery Platforms Coupled With Luminex Multiple Analyte Profiling Technology
Source: Front Cell Dev Biol. 2020 Jun 19;8:444. doi: 10.3389/fcell.2020.00444 (PMC7317666; doi:10.3389/fcell.2020.00444)
Supplement: TABLE S1 — The list of 263 upstream regulators identified based on the IPA core analysis of the molecular targets of S1P. [file Table_1.docx]

**Supplementary Table 1:** The list of 263 upstream regulators identified based on the IPA core analysis of the molecular targets of S1P

| **Upstream regulator** | **Molecule type** | **p-value of overlap** | **Target molecules in the dataset** |
| --- | --- | --- | --- |
| Camk | complex | 0.0000191 | KDR,S1PR3 |
| PDGF BB | complex | 0.0452 | MMP12,PIM1 |
| Ap1 | complex | 0.014 | C3AR1,MMP2 |
| NFkB (complex) | complex | 0.0221 | ERAP1,ERAP2,MMP2,SOAT1 |
| LDL | complex | 0.00000356 | CASP3,CETP,ECE1,MMP2,SPHK1 |
| IgG | complex | 0.00357 | CTSC,MMP12,PIM1,ST14 |
| BCR (complex) | complex | 0.0323 | PIM1,S1PR1 |
| Immunoglobulin | complex | 0.0115 | C3AR1,SPHK1 |
| Complement | complex | 0.0175 | CASP1 |
| CSF1 | cytokine | 0.00767 | CTSK,MMP12 |
| IL5 | cytokine | 0.0197 | CTSC,PIM1 |
| IL4 | cytokine | 0.00132 | CYSLTR1,IDO1,PIM1,ST14 |
| IL21 | cytokine | 0.0296 | IDO1,MMP2 |
| CSF2 | cytokine | 0.0133 | CASP3,CTSC,PIM1 |
| TNF | cytokine | 0.00118 | CTSC,GSTA1,IDO1,MMP12,MMP2,MMP8,NR3C1,SOAT1 |
| CRH | cytokine | 0.0175 | TPH1 |
| IL13 | cytokine | 0.0000174 | C3AR1,CASP1,CTSC,CYSLTR1,ENPP2,LTA4H,ST14 |
| IFNB1 | cytokine | 0.000528 | CASP1,CASP3,IDO1 |
| TIMP1 | cytokine | 0.0389 | MME |
| LIF | cytokine | 0.0131 | ERAP1 |
| IFNG | cytokine | 0.000145 | ACE,CASP1,CASP3,ERAP2,IDO1,MMP2,PIM1,SOAT1 |
| IL1B | cytokine | 0.0236 | GSTA1,KDR,MMP12,MMP8 |
| NSD3 | enzyme | 0.0131 | PIM2 |
| HSPA6 | enzyme | 0.0044 | SPHK1 |
| TUT1 | enzyme | 0.0261 | KISS1R |
| SRSF9 | enzyme | 0.0044 | NR3C1 |
| HIF1AN | enzyme | 0.0346 | CA9 |
| XRCC6 | enzyme | 0.0175 | SIRT1 |
| DICER1 | enzyme | 0.00047 | AKT1,ANPEP,KDR,S1PR1 |
| NOS3 | enzyme | 0.0131 | GSTP1 |
| TGM2 | enzyme | 0.0121 | CA2,MMP2,MMP8,PIM1 |
| FHIT | enzyme | 0.0304 | MMP2 |
| PTGS1 | enzyme | 0.00877 | MMP2 |
| SOD3 | enzyme | 0.0131 | CA9 |
| ASAH1 | enzyme | 0.0058 | SPHK1,SPHK2 |
| RNF19A | enzyme | 0.0044 | CASR |
| RNF139 | enzyme | 0.00877 | FDFT1 |
| UGCG | enzyme | 0.0346 | CASP3 |
| APEX1 | enzyme | 0.0175 | SIRT1 |
| BIRC7 | enzyme | 0.00877 | CASP3 |
| LTB4R2 | G-protein coupled receptor | 0.0175 | MMP2 |
| CYSLTR2 | G-protein coupled receptor | 0.00877 | CYSLTR1 |
| CCKBR | G-protein coupled receptor | 0.00877 | MAPK1 |
| LPAR1 | G-protein coupled receptor | 0.000526 | S1PR3,SPHK1 |
| GABBR2 | G-protein coupled receptor | 0.0131 | CASR |
| GABBR1 | G-protein coupled receptor | 0.0044 | CASR |
| Alpha 1 antitrypsin | group | 0.00877 | MMP2 |
| Creb | group | 0.0287 | MMP2,NR3C1 |
| Ctbp | group | 0.0431 | SIRT1 |
| Wnt | group | 0.0431 | KDR |
| Rb | group | 0.00311 | DHFR,TYMS |
| Calmodulin | group | 0.0000571 | KDR,S1PR3 |
| Beta Secretase | group | 0.0131 | MME |
| Mek | group | 0.00375 | DNMT3B,HRH1,KDR,MMP2 |
| Vegf | group | 0.0346 | KDR |
| ETS | group | 0.0304 | C3AR1 |
| Akt | group | 0.00778 | EZH2,MMP12,MMP2 |
| DNAJ | group | 0.0044 | ENPP2 |
| Interferon alpha | group | 0.0184 | CASP1,CASP3,ENPP2,IDO1 |
| Histone h4 | group | 0.00234 | DHFR,SPHK1,TYMS |
| Eotaxin | group | 0.0131 | MAPK1 |
| Gamma tubulin | group | 0.0131 | CASP3 |
| PI3K (family) | group | 0.00977 | MMP12,MMP2,SIRT1 |
| TCF | group | 0.0051 | IDO1,MME,MMP2 |
| FGF16 | growth factor | 0.0175 | MMP2 |
| IGF2 | growth factor | 0.0389 | MMP12 |
| VEGFA | growth factor | 0.000775 | KDR,MMP12,MMP2 |
| VEGFB | growth factor | 0.0044 | MMP12 |
| TGFB1 | growth factor | 0.0195 | KDR,MAPK1,MMP12,MMP2,SPHK1 |
| VEGFD | growth factor | 0.0175 | MMP12 |
| HGF | growth factor | 0.000000328 | AKT1,CA9,CTSK,MAPK14,MMP2,MMP8,ST14 |
| TRPC1 | ion channel | 0.00123 | KDR,S1PR3 |
| KCNMA1 | ion channel | 0.0131 | MMP2 |
| OBSCN | kinase | 0.0175 | CASP3 |
| PIP5K1A | kinase | 0.0346 | KISS1R |
| EIF2AK3 | kinase | 0.0473 | CA9 |
| PRKCD | kinase | 0.00246 | HRH1,MMP12,MMP2,SPHK1 |
| GIT1 | kinase | 0.0175 | MMP2 |
| PRKCE | kinase | 0.00923 | DLG4,PIM1 |
| ULK1 | kinase | 0.00877 | CASP3 |
| MTOR | kinase | 0.00646 | CASP1,SOAT1,ZAP70 |
| CDK5R1 | kinase | 0.0175 | MMP2 |
| AXL | kinase | 0.0261 | MMP2 |
| MET | kinase | 0.0000312 | AKT1,MMP2,PIM1,PIM3 |
| DDR2 | kinase | 0.0346 | MMP2 |
| CDK6 | kinase | 0.0131 | DHFR |
| CARD11 | kinase | 0.0175 | MME |
| PRKCB | kinase | 0.0218 | MMP2 |
| ERBB2 | kinase | 0.00909 | AKT1,DHFR,GLO1,GSK3B,TYMS |
| FLT3 | kinase | 0.0389 | PIM1 |
| TGFBR2 | kinase | 0.00321 | CASP1,MMP2,PRSS1 |
| NR1H4 | ligand-dependent nuclear receptor | 0.0127 | CETP,NR3C1 |
| ESR2 | ligand-dependent nuclear receptor | 0.0153 | GSTP1,MME |
| NR2F2 | ligand-dependent nuclear receptor | 0.0431 | KDR |
| miR-515-5p (and other miRNAs w/seed UCUCCAA) | mature microRNA | 0.0175 | SPHK1 |
| miR-148a-3p (and other miRNAs w/seed CAGUGCA) | mature microRNA | 0.0389 | DNMT3B |
| miR-34a-5p (and other miRNAs w/seed GGCAGUG) | mature microRNA | 0.00978 | DHFR,SIRT1 |
| miR-200a-5p (and other miRNAs w/seed AUCUUAC) | mature microRNA | 0.0175 | EZH2 |
| miR-181a-5p (and other miRNAs w/seed ACAUUCA) | mature microRNA | 0.0473 | EZH2 |
| miR-451a (and other miRNAs w/seed AACCGUU) | mature microRNA | 0.00084 | AKT1,MMP2 |
| miR-101-3p (and other miRNAs w/seed ACAGUAC) | mature microRNA | 0.0304 | EZH2 |
| miR-532-3p (miRNAs w/seed CUCCCAC) | mature microRNA | 0.0175 | AKT1 |
| miR-491-5p (and other miRNAs w/seed GUGGGGA) | mature microRNA | 0.0304 | MMP2 |
| miR-1-3p (and other miRNAs w/seed GGAAUGU) | mature microRNA | 0.0131 | SPHK1 |
| miR-26a-5p (and other miRNAs w/seed UCAAGUA) | mature microRNA | 0.00168 | EZH2,GSK3B |
| mir-296 | microRNA | 0.000283 | CA9,PRSS1 |
| mir-637 | microRNA | 0.0218 | AKT1 |
| mir-185 | microRNA | 0.0346 | FDFT1 |
| mir-181 | microRNA | 0.00249 | EZH2,MME |
| mir-203 | microRNA | 0.0431 | EZH2 |
| mir-8 | microRNA | 0.000892 | AKT1,EZH2,KDR |
| mir-374 | microRNA | 0.00877 | AKT1 |
| mir-1180 | microRNA | 0.0000571 | AKT1,GSK3B |
| MIR585 | microRNA | 0.00877 | CASP3 |
| BDNF-AS | other | 0.0044 | CASP3 |
| UCA1 | other | 0.0218 | EZH2 |
| CYTOR | other | 0.0261 | MAPK14 |
| LINC01139 | other | 0.0431 | MAPK1 |
| PDLIM2 | other | 0.0071 | ENPP2,GSTM1,MME |
| RNU1-1 | other | 0.0131 | IDO1 |
| MYOC | other | 0.00308 | CA2,CHRM3,PTGER4 |
| NAALADL2 | other | 0.0389 | S1PR3 |
| BTBD7 | other | 0.00877 | MMP2 |
| MEMO1 | other | 0.0346 | CA2 |
| DGCR5 | other | 0.000114 | AKT1,GSK3B |
| ZMYND10 | other | 0.0261 | MMP2 |
| SNHG20 | other | 0.0473 | GSK3B |
| S100A14 | other | 0.0044 | MMP2 |
| IFT88 | other | 0.0473 | FDPS |
| CABIN1 | other | 0.00877 | MMP2 |
| KCNQ1OT1 | other | 0.0218 | CASP1 |
| DEF6 | other | 0.0431 | MMP2 |
| CD81 | other | 0.0261 | MMP2 |
| L1CAM | other | 0.0304 | MMP2 |
| SHC1 | other | 0.000528 | GSTP1,S1PR1,S1PR2 |
| SPOP | other | 0.0346 | FDFT1 |
| CGB3 (includes others) | other | 0.0044 | MMP2 |
| TIMP2 | other | 0.0131 | MMP2 |
| COL18A1 | other | 0.000204 | F10,KDR,MAPK1,MMP2 |
| TPM3 | other | 0.0389 | MMP2 |
| TAC4 | other | 0.00877 | MMP2 |
| RASSF1 | other | 0.0473 | MMP2 |
| ELAVL1 | other | 0.0473 | DGAT1,SIRT1 |
| FBLN2 | other | 0.0131 | MMP2 |
| MYOF | other | 0.0044 | KDR |
| ATG5 | other | 0.0304 | CASP3 |
| KISS1 | other | 0.00877 | GSTP1 |
| HIF1A-AS1 | other | 0.00877 | CASP3 |
| CLDN7 | other | 0.0163 | CA12,LTA4H,MMP2 |
| GAS5 | other | 0.0389 | CASP3 |
| CD209 | other | 0.0473 | MMP2 |
| BANCR | other | 0.0304 | MMP2 |
| FSTL1 | other | 0.00877 | CASP1 |
| XAF1 | other | 0.0175 | CASP3 |
| TIA1 | other | 0.0389 | SIRT1 |
| TERC | other | 0.0175 | CASP1 |
| RBL2 | other | 0.0067 | DHFR,TYMS |
| OCLN | other | 0.000395 | MMP2,SIRT1 |
| HNRNPA2B1 | other | 0.00339 | CA12,LPAR1,PRSS1,S1PR3 |
| ITGB6 | other | 0.0131 | MMP2 |
| CELF2 | other | 0.0175 | SIRT1 |
| TNC | other | 0.0346 | MMP2 |
| CLU | other | 0.0022 | AKT1,MMP2 |
| SCUBE3 | other | 0.0346 | MMP2 |
| LSINCT5 | other | 0.0175 | CASP1 |
| GRB10 | other | 0.0175 | MMP12 |
| THY1 | other | 0.0304 | MMP2 |
| LGALS3 | other | 0.0000585 | CASP3,ENPP2,GSK3B,PRSS1 |
| CCNA1 | other | 0.0131 | MMP2 |
| MCAM | other | 0.0389 | MMP2 |
| SPINT2 | other | 0.0473 | ST14 |
| NEDD9 | other | 0.0121 | CA9,MMP2 |
| SRSF5 | other | 0.0131 | NR3C1 |
| UCA1 | other | 0.0389 | CASP3 |
| SENP7 | peptidase | 0.0304 | DHFR |
| UCHL5 | peptidase | 0.0304 | CASP3 |
| CASP1 | peptidase | 0.0175 | KDR |
| PROC | peptidase | 0.0218 | MMP2 |
| PRSS2 | peptidase | 0.00877 | MMP8 |
| F10 | peptidase | 0.0261 | MMP2 |
| SENP1 | peptidase | 0.00877 | MMP2 |
| ELANE | peptidase | 0.0389 | MMP2 |
| CAPNS1 | peptidase | 0.0044 | MMP2 |
| PPP1R1B | phosphatase | 0.0473 | CASP3 |
| PPP2CA | phosphatase | 0.0473 | CASP1 |
| PTPRR | phosphatase | 0.0261 | DNMT3B |
| IGBP1 | phosphatase | 0.0131 | CASP1 |
| PTPN9 | phosphatase | 0.0044 | MMP2 |
| ARHGAP35 | transcription regulator | 0.0044 | KDR |
| RRP1B | transcription regulator | 0.0431 | CASP3 |
| ZNF24 | transcription regulator | 0.0175 | MMP2 |
| MEN1 | transcription regulator | 0.00877 | AKT1 |
| STAT3 | transcription regulator | 0.000209 | AKT1,CA9,CASP3,DNMT3B,MMP2,PIM1 |
| E2F1 | transcription regulator | 0.00166 | CASP3,DHFR,EZH2,KDR,TYMS |
| TFAP2A | transcription regulator | 0.0212 | GLO1,MMP2 |
| HDAC10 | transcription regulator | 0.0175 | MMP2 |
| HTATIP2 | transcription regulator | 0.0346 | MMP2 |
| RUNX3 | transcription regulator | 0.0473 | AKT1 |
| EPAS1 | transcription regulator | 0.00801 | CA9,KDR,SPHK1 |
| RBL1 | transcription regulator | 0.0067 | DHFR,TYMS |
| PAX8 | transcription regulator | 0.0389 | DHFR |
| HIF1A | transcription regulator | 0.0159 | CA9,MMP2,PIM2,SPHK1 |
| CEBPA | transcription regulator | 0.0185 | ANPEP,C3AR1,CA2 |
| IRF2 | transcription regulator | 0.0421 | CA12,ERAP1 |
| EED | transcription regulator | 0.0346 | MMP2 |
| TWIST1 | transcription regulator | 0.0182 | CASP3,MMP2 |
| JUN | transcription regulator | 0.00385 | C3AR1,GSTP1,MMP2,SPHK1 |
| TIAL1 | transcription regulator | 0.0175 | SIRT1 |
| MBD2 | transcription regulator | 0.0147 | GSTP1,MMP2 |
| POU5F1 | transcription regulator | 0.00282 | AKT1,CASP1,CASP3,MMP2 |
| CDKN2B | transcription regulator | 0.0000571 | DHFR,DNMT3B |
| FOXM1 | transcription regulator | 0.0441 | KDR,MMP2 |
| TP73 | transcription regulator | 0.0391 | CASP3,DHFR,SPHK2 |
| HHEX | transcription regulator | 0.0175 | KDR |
| EZH2 | transcription regulator | 0.00759 | CHRM3,MMP2,PIM2,SIRT1 |
| EP300 | transcription regulator | 0.00566 | CA12,SIRT1,SPHK1 |
| IRF1 | transcription regulator | 0.00147 | CASP1,ERAP1,IDO1 |
| MTA1 | transcription regulator | 0.0022 | GSK3B,SIRT1 |
| BCL6B | transcription regulator | 0.0431 | CASP3 |
| SMARCA2 | transcription regulator | 0.000407 | DHFR,KDR,TYMS |
| E2F4 | transcription regulator | 0.032 | DHFR,FDFT1,LPAR1 |
| NKX2-5 | transcription regulator | 0.00877 | ECE1 |
| RB1 | transcription regulator | 0.00755 | DHFR,KDR,TYMS |
| SMAD3 | transcription regulator | 0.038 | LPAR1,S1PR3 |
| YAP1 | transcription regulator | 0.0228 | CASP3,TYMS |
| DMTF1 | transcription regulator | 0.0044 | ANPEP |
| SIN3B | transcription regulator | 0.00249 | DHFR,TYMS |
| GTF3A | transcription regulator | 0.00877 | PIM1 |
| HTT | transcription regulator | 0.0304 | CASP1 |
| GTF2I | transcription regulator | 0.0131 | KDR |
| SMARCC2 | transcription regulator | 0.0175 | KDR |
| PRDM1 | transcription regulator | 0.0022 | ERAP1,IDO1 |
| RELA | transcription regulator | 0.0426 | ERAP1,ERAP2,GSTA1 |
| BTG1 | transcription regulator | 0.00877 | NR3C1 |
| FOS | transcription regulator | 0.00378 | C3AR1,GSTP1,MMP8 |
| HEY1 | transcription regulator | 0.0304 | KDR |
| HDAC1 | transcription regulator | 0.000369 | DHFR,GSTP1,MME,TYMS |
| EHF | transcription regulator | 0.041 | ANPEP,KDR |
| DAXX | transcription regulator | 0.0218 | CASP3 |
| SMARCA4 | transcription regulator | 0.0144 | CASP1,GSTP1,KDR,MAPK1,MMP2,SPHK1 |
| CTNNB1 | transcription regulator | 0.00836 | CA9,IDO1,MME,MMP2 |
| FOXN3 | transcription regulator | 0.0261 | PIM2 |
| BHLHE41 | transcription regulator | 0.0261 | CA9 |
| HIC1 | transcription regulator | 0.000141 | CA12,CA2,MMP12,SIRT1 |
| TP53 | transcription regulator | 0.00000173 | AKT1,CASP1,DHFR,FDFT1,FDPS,GSK3B,KDR,MAPK1,MMP2,PIM1 |
| PROX1 | transcription regulator | 0.0346 | KDR |
| SMARCC1 | transcription regulator | 0.0131 | KDR |
| RPSA | translation regulator | 0.0175 | MMP2 |
| JMJD6 | transmembrane receptor | 0.0131 | PIM2 |
| IL13RA2 | transmembrane receptor | 0.0175 | MMP12 |
| TREM1 | transmembrane receptor | 0.0385 | IDO1,PIM2,S1PR3 |
| CD4 | transmembrane receptor | 0.0346 | MME |
| TNFRSF11A | transmembrane receptor | 0.0261 | MME |
| ITGA6 | transmembrane receptor | 0.0431 | ENPP2 |
| ENG | transmembrane receptor | 0.0473 | SIRT1 |
| TLR9 | transmembrane receptor | 0.0314 | AKT1,ZAP70 |
| PRLR | transmembrane receptor | 0.0218 | EZH2 |
| CHRNA3 | transmembrane receptor | 0.0431 | CASP3 |
| IFITM1 | transmembrane receptor | 0.00279 | MAPK1,MMP12 |
| SLC20A1 | transporter | 0.0044 | AKT1 |
| SLC2A1 | transporter | 0.0044 | MMP2 |
| FOLR1 | transporter | 0.00877 | TYMS |
| APOE | transporter | 0.00492 | CASP1,SIRT1,SLC6A1 |
