# Supplementary material for: Decoding the Role of Sphingosine-1-Phosphate in Asthma and Other Respiratory System Diseases Using Next Generation Knowledge Discovery Platforms Coupled With Luminex Multiple Analyte Profiling Technology
Source: Front Cell Dev Biol. 2020 Jun 19;8:444. doi: 10.3389/fcell.2020.00444 (PMC7317666; doi:10.3389/fcell.2020.00444)
Supplement: TABLE S2 — Simple linear regression analysis of S1P with upstream regulatory cytokines and growth factors in asthma patients. [file Table_2.docx]

**Supplementary Table 2:** Simple linear regression analysis of S1P with upstream regulatory cytokines and growth factors in asthma patients.

| **Simple linear regression** | **CSF2** | **HGF** | **VEGF** | **TNFa** | **IL-1b** | **IFN-g** | **IL-4** | **IL-5** | **IL-13** |
| --- | --- | --- | --- | --- | --- | --- | --- | --- | --- |
| Best-fit values |  |  |  |  |  |  |  |  |  |
| Slope | 49.83 | 599.5 | 15.85 | 110.9 | 1.453 | 17.26 | 77.59 | 23.27 | 57.52 |
| Y-intercept | 6.943 | -155.4 | -2.158 | 37.13 | 0.2732 | 67.22 | 19.66 | -1.241 | 29.27 |
| X-intercept | -0.1393 | 0.2593 | 0.1362 | -0.3349 | -0.188 | -3.895 | -0.2534 | 0.05331 | -0.5089 |
| 1/slope | 0.02007 | 0.001668 | 0.06311 | 0.009018 | 0.6881 | 0.05795 | 0.01289 | 0.04297 | 0.01738 |
|  |  |  |  |  |  |  |  |  |  |
| Std. Error |  |  |  |  |  |  |  |  |  |
| Slope | 14.63 | 155.6 | 3.12 | 22.83 | 0.3889 | 3.911 | 19.98 | 5.538 | 13.47 |
| Y-intercept | 14.87 | 158 | 3.169 | 23.2 | 0.3951 | 3.973 | 20.29 | 5.625 | 13.69 |
|  |  |  |  |  |  |  |  |  |  |
| 95% Confidence Intervals |  |  |  |  |  |  |  |  |  |
| Slope | 17.22 to 82.43 | 252.9 to 946.1 | 8.895 to 22.80 | 60.01 to 161.8 | 0.5868 to 2.320 | 8.542 to 25.97 | 33.08 to 122.1 | 10.93 to 35.61 | 27.50 to 87.55 |
| Y-intercept | -26.18 to 40.06 | -507.5 to 196.6 | -9.219 to 4.904 | -14.55 to 88.82 | -0.6070 to 1.154 | 58.36 to 76.07 | -25.56 to 64.88 | -13.77 to 11.29 | -1.225 to 59.77 |
| X-intercept | -2.262 to 0.3267 | -0.7488 to 0.5570 | -0.5380 to 0.4144 | -1.455 to 0.09148 | -1.918 to 0.2682 | -8.860 to -2.259 | -1.918 to 0.2140 | -1.005 to 0.3975 | -2.137 to 0.01423 |
|  |  |  |  |  |  |  |  |  |  |
| Goodness of Fit |  |  |  |  |  |  |  |  |  |
| R squared | 0.5369 | 0.5976 | 0.7206 | 0.7022 | 0.5827 | 0.6606 | 0.6013 | 0.6385 | 0.6457 |
| Sy.x | 15.83 | 168.2 | 3.374 | 24.7 | 0.4206 | 4.23 | 21.61 | 5.989 | 14.57 |
|  |  |  |  |  |  |  |  |  |  |
| Is slope significantly non-zero? |  |  |  |  |  |  |  |  |  |
| F | 11.59 | 14.85 | 25.8 | 23.58 | 13.97 | 19.47 | 15.08 | 17.66 | 18.23 |
| DFn | 1 | 10 | 1 | 10 | 1 | 1 | 10 | 1 | 10 |
| P value | 0.0067 | 0.0032 | 0.0005 | 0.0007 | 0.0039 | 0.0013 | 0.003 | 0.0018 | 0.0016 |
| Deviation from zero? | Significant | Significant | Significant | Significant | Significant | Significant | Significant | Significant | Significant |
|  |  |  |  |  |  |  |  |  |  |
| Equation | Y = 49.83*X + 6.943 | Y = 599.5*X - 155.4 | Y = 15.85*X - 2.158 | Y = 110.9*X + 37.13 | Y = 1.453*X + 0.2732 | Y = 17.26*X + 67.22 | Y = 77.59*X + 19.66 | Y = 23.27*X - 1.241 | Y = 57.52*X + 29.27 |
|  |  |  |  |  |  |  |  |  |  |
| Data |  |  |  |  |  |  |  |  |  |
| Number of X values | 12 | 12 | 12 | 12 | 12 | 12 | 12 | 12 | 12 |
| Maximum number of Y replicates | 1 | 1 | 1 | 1 | 1 | 1 | 1 | 1 | 1 |
| Total number of values | 12 | 12 | 12 | 12 | 12 | 12 | 12 | 12 | 12 |
| Number of missing values | 0 | 0 | 0 | 0 | 0 | 0 | 0 | 0 | 0 |
